# Characterization of mRNA profiles of the exosome-like vesicles in porcine follicular fluid

**DOI:** 10.1371/journal.pone.0217760

**Published:** 2019-06-12

**Authors:** Yuta Matsuno, Takuya Kanke, Natsumi Maruyama, Wataru Fujii, Kunihiko Naito, Koji Sugiura

**Affiliations:** Laboratory of Applied Genetics, Department of Animal Resource Sciences, Graduate School of Agricultural and Life Sciences, The University of Tokyo, Tokyo, Japan; Chuo University, JAPAN

## Abstract

Extracellular vesicles such as exosomes contain several types of transcripts, including mRNAs and micro RNAs (miRNAs), and have emerged as important mediators of cell-to-cell communication. Exosome-like vesicles were identified in the ovarian follicles of several mammalian species. Although the miRNA contents have been extensively characterized, the detailed investigation of their mRNA profiles is lacking. Here, we characterize the mRNA profiles of exosome-like vesicles in ovarian follicles in a pig model. The mRNA contents of the exosome-like vesicles isolated from porcine follicular fluid were analyzed and compared with those from mural granulosa cells (MGCs) using the Illumina HiSeq platform. Bioinformatics studies suggested that the exosomal mRNAs are enriched in those encoding proteins involved in metabolic, phosphatidylinositol-4,5-bisphosphate 3-kinase (PI3K) -protein kinase B (AKT), and mitogen-activated protein kinase (MAPK) pathways. While the mRNA profile of the exosome-like vesicles resembled that of MGCs, the vesicles contained mRNAs barely detectable in MGCs. Thus, while the majority of the vesicles are likely to be secreted from MGCs, some may originate from other cell types, including theca cells and oocytes, as well as the cells of non-ovarian organs/tissues. Therefore, the mRNA profiles unveiled several novel characteristics of the exosome-like vesicles in ovarian follicles.

## Introduction

Exosomes are lipid bilayer vesicles of around 40–200 nm diameter produced by most cell types [1]. These vesicles contain several bioactive materials such as proteins, lipids, micro RNAs (miRNAs), and mRNAs with properties slightly different from their originating cells [2, 3]. Exosomes mediate cell-to-cell communication by transferring these molecules to target cells, wherein the transferred molecules may affect multiple biological processes [4–6]. Exosomes produced from several organs and tissues are readily detectable in the blood stream and may be considered as potential diagnostic markers of diseases such as cancers [7–9].

The presence of exosome-like vesicles in ovarian follicular fluids was first reported in mares [10]. Exosome-like vesicles isolated from equine follicular fluid contained several proteins and miRNAs, and were taken-up by granulosa cells both in vitro and in vivo. The miRNAs detected were predicted to target several signaling pathways, including the WNT, transforming growth factor beta (TGFβ), and mitogen-activated protein kinase (MAPK) pathways [10, 11]. These pathways are involved in the regulation of ovarian functions, including folliculogenesis, luteogenesis, and steroidogenesis [12–15]. The presence of exosome-like vesicles was also reported in follicular fluids of several other mammalian species, including human, bovine, and pig [16–19]. The miRNA content in the exosome-like vesicles from human and bovine has been studied [16, 17], and these miRNAs are known to target signaling pathways similar to those targeted by equine vesicles. Therefore, these vesicles may play a critical role in the regulation of ovarian functions via miRNA transfer [20].

In general, exosomes contain many other molecules aside from miRNAs, such as mRNAs, that can be delivered and translated into proteins in recipient cells [2, 21, 22]. The exosome-mediated transfer of mRNAs was first reported by Valadi and colleagues who demonstrated that exosomes are capable of shuttling mRNAs between mast cells [2]. In addition, cancer-derived exosomes carry matrix metalloproteinase 1 (*MMP1*) mRNA to induce apoptosis in recipient mesothelial cells [21]. Exosomes can deliver the mRNA encoding Cre recombinase to recipient cells to perform Cre-LoxP-mediated recombination in vivo and in vitro [22]. Therefore, mRNAs are also thought to be critical mediators of exosomal functions.

The miRNA contents of follicular exosome-like vesicles have been extensively characterized, and these studies provide an insight in the function of the vesicles during folliculogenesis in mammals; however, no report has described the complete characterization of the mRNA contents of exosome-like vesicles. Therefore, in the present study, we evaluated the characteristics of transcriptomic profile of mRNA content in exosome-like vesicles from ovarian follicles using pig as a model. The presence of the exosome-like vesicles in porcine follicular fluid (pFF) has been previously reported [19]. As mural granulosa cells (MGCs) are one of the most abundant cell types in ovarian follicles, the transcriptomic profile of the MGCs was also investigated to test whether MGCs are the main producers of the follicular exosome-like vesicles.

## Materials and methods

### Collection of pFF and MGCs

Porcine ovaries of prepubertal gilts were collected at a commercial slaughterhouse (Tokyo Shibaura Zouki, Co., Ltd., Tokyo, Japan) and transported to our laboratory at approximately 37°C in saline. pFF was collected from antral follicles (2–5 mm in diameter), as previously reported [23]. Great care was taken to avoid contamination of the fluid with blood.

MGCs were collected from antral follicles (2–5 mm in diameter), as previously reported [19].

### Isolation of exosome-like vesicles from pFF

The fraction containing exosome-like vesicles was isolated from pFF, as previously reported [19]. In brief, pFF was first centrifuged at 2,000 ×*g* and 4°C for 30 min, followed by another step of centrifugation at 12,000 ×*g* and 4°C for 45 min. The sample was filtered through a 0.22-μm membrane (Merck Millipore, Darmstadt, Germany) to remove cells and debris. After the filtration, the exosomal fraction was extracted using a Total Exosome Isolation (from serum) reagent (Life Technologies, Inc. Carlsbad, CA, USA). The samples were immediately subjected to the next procedure.

### Transmission electron microscopy observations of the exosomal fraction

The exosomal fraction isolated from pFF was observed with a transmission electron microscope (JEM-1010; JEOL, Tokyo, Japan) as previously reported [19].

### Western blotting analysis

Western blotting analysis was conducted as previously reported [19]. The primary antibodies used were anti-HSC70 antibody (MAB2191; Abnova, Taipei, Taiwan), anti-CD63 antibody (sc-5725; Santa Cruz Biotechnology, Texas, USA), and anti-CYCS antibody (sc-13156), and the secondary antibodies used were horseradish peroxidase conjugated anti-rat IgG antibodies (81–9520; Invitrogen) and anti-mouse IgG antibodies (115-035-044; Jackson ImmunoResearch, West Grove, PA, USA). Signals were visualized using an Immunostar LD Kit (Wako, Tokyo, Japan) and the C-DiGit Blot Scanner and Image Studio for C-DiGit (LI-COR, Lincoln, NE, USA) according to the manufacturer’s protocols.

### RNA profiling using a bioanalyzer

Total RNA was extracted from the exosomal fraction and MGCs using a ReliaPrep Cell Miniprep System (Promega K.K., Tokyo, Japan). The total RNA profiles were visualized by using an Agilent 2100 bioanalyzer (Agilent Technologies, Palo Alto, CA, USA) with Agilent RNA6000 Pico Kit (Agilent Technologies) according to the manufacturer’s protocols.

### RNA sequencing with Illumina HiSeq platform

The total RNA was extracted from three biologically independent samples of the exosomal fractions and MGCs, respectively, using a ReliaPrep Cell Miniprep System (Promega K.K.). Library construction, quality control, and sequencing were performed by Filgen Inc. (Aichi, Japan). A total amount of 3 μg of RNA per sample was used as an input material for the RNA sample preparations. mRNA was purified from total RNA using poly-T oligo-attached magnetic beads. Sequencing libraries were generated using NEBNext Ultra RNA Library Prep Kit for Illumina (NEB, USA). The library preparations were sequenced on an Illumina HiSeq platform, and 150 base paired end reads were generated. The sequenced reads (raw reads) were subjected to several quality checks. In this step, clean data (clean reads) were obtained by removing reads containing adapter and poly-N (N represents the undetermined base) as well as low quality reads from raw data. The filtering process was as follows: (1) Remove reads containing adapters, (2) remove reads containing N > 10%, and (3) remove reads containing low-quality (Q score ≤ 5) base, which was over 50% of the total base. The data have been deposited in the Data Bank of Japan (DDBJ, http://www.ddbj.nig.ac.jp, data set DRA008080).

All the downstream analyses were based on the clean data using CLC Genomics Workbench version 11.0.1 (QIAGEN K.K., Tokyo, Japan) with its default parameters. Clean reads were trimmed to remove low-quality sequence (limit = 0.05) and ambiguous nucleotides (maximal two nucleotides allowed). The proceeded reads were aligned with the Sscrofa11.1 porcine genome annotated with genes and transcripts and to generate the gene expression values in the normalized form of reads per kilobase per million mapped reads (RPKM) [24]. The criteria used to determine the detected transcripts was an average RPKM value of more than 0.1 for three replicates.

### Reverse-transcription and polymerase chain reaction (RT-PCR)

The total RNA was extracted from the exosomal fraction and MGCs using a ReliaPrep Cell Miniprep System. Total RNA was reverse transcribed using a ReverTraAce qPCR Master Mix with gDNA Remover (Toyobo, Osaka, Japan), and PCR was performed using a BIOTAQ DNA polymerase (Bioline Ltd., London, UK). Samples were denatured for 2 min at 95°C and incubated for 35 cycles under the following conditions: 95°C for 30 s, 55°C for 30 s, and 72°C for 2 min, followed by the final elongation for 5 min at 72°C. The PCR products were subjected to agarose gel electrophoresis. The PCR primers used were as follows: 5’-TTTTTCGCAACGGGTTTGCC-3’ and 5’-TGTGACAGATTTTTGGTCAAGTTGT-3’ for eukaryotic translation elongation factor 1 alpha 1 (*EEF1A1*; NM_001003662.1); 5’-GACTCCGCCTCTCAGCTATC-3’ and 5’-GCTTGAGTGTGAGCCTTTCG-3’ for ferritin light chain (*FTL*; NM_001244131.1).

### RNA degradation analysis

2 μg/mL RNase A (NIPPON GENE Co., Ltd., Tokyo, Japan) and 2% Triton X-100 (Sigma-Aldrich Japan K.K. Tokyo, Japan) was added into pFF, and incubated at 37°C for 20 min. As a control treatment, the equal volume of phosphate buffered saline (PBS) was added to pFF instead of the reagent and incubated at 37°C for 20 min. After the incubation, pFF was subjected into the exosome-like vesicle isolation procedure, then RNA was extracted from the exosomal fractions as described above.

The effectiveness of RNase A treatment was examined by Real-time PCR reactions. Real-time PCR reactions were performed using a THUNDERBIRD qPCR Mix (Toyobo) and an ABI Step One Plus real-time PCR system (Applied Biosystems) according to the manufacturer’s protocols. The PCR primers used were as follows: 5’-ATGCGGTGGGATCGACAAAA-3’ and 5’-AGTTTGTCCAAGACCCAGGC-3’ for *EEF1A1*; 5’-GAAAATGCAAAACCAGCGCG-3’ and 5’-CTTCCATAGCGTCCTGGGTT-3’ for *FTL*. To avoid false-positive signals, dissociation-curve analyses were performed at the end of the analyses, and the PCR products were subjected to agarose gel electrophoresis to confirm the single amplification and sizes of the products.

### Data analysis

The Database for Annotation, Visualization and Integrated Discovery (DAVID) bioinformatics resources was used for pathway analysis [25, 26]. Principal component analysis (PCA) was performed using the CLC Genomics Workbench, with the data sets of porcine tissue transcriptomes downloaded from NCBI GEO (S1 Table). The downloaded raw data were subjected to the filtering procedure before using for PCA. In addition to literature, tissue specificity of transcripts was investigated with PaGenBase [27] (http://bioinf.xmu.edu.cn/PaGenBase/index.jsp) by referring to the data sets for human [28–34], as porcine data sets were unavailable.

All experiments were repeated at least three times. Statistical analyses were conducted using Microsoft Excel (Microsoft) and the program Excel-Statistics (Social Survey Research Information Co., Ltd., Tokyo, Japan). The Tukey-Kramer test was used for multiple comparisons. A *P*-value < 0.05 was considered statistically significant.

## Results

### Validation of exosome-like vesicles in the exosomal fraction isolated from pFF

**Fig 1:**
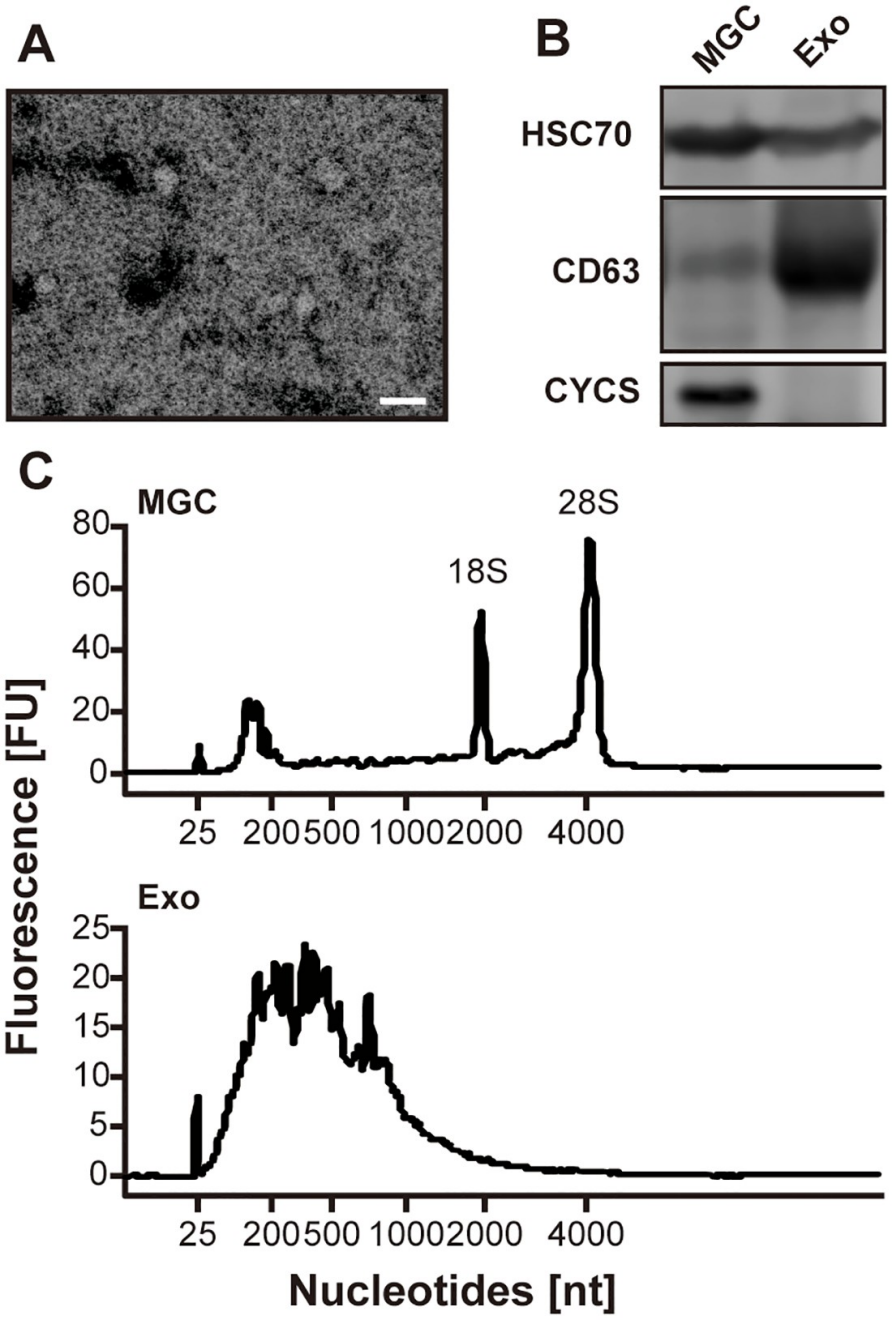
Detection of exosome-like vesicles in the exosomal fraction isolated from pFF. (A) Representative photograph of vesicles in the exosomal fraction isolated from pFF observed using transmission electron microscopy. The scale bar indicates 100 nm. (B) Western blotting analysis for HSC70, CD63, and CYCS. MGC, mural granulosa cell; Exo, exosomal fraction. (C) Representative electropherograms observed using bioanalyzer. FU, fluorescence intensity units; nt, nucleotides.

To validate the detection of exosome-like vesicles in pFF, we performed the electron microscopy observation (TEM), western blotting analysis, and bioanalyzer analysis. TEM observation demonstrated that round-shaped vesicles about 100 nm in diameter were observed in the exosomal fraction (Fig 1A). With western blotting analysis, well-known exosomal makers, CD63 molecule (CD63) and heat shock protein 70 (HSC70) [35], were detected in both the exosomal fraction and MGCs. On the other hand, cytochrome C (CYCS) was readily detected in the MGCs, but it was absent in the exosomal fraction, indicating that the exosomal fraction was not contaminated with apoptotic bodies or cell debris (Fig 1B). In addition, bioanalyzer analysis demonstrated that the RNAs isolated from the exosomal fraction were enriched in small RNAs, and the peaks of ribosomal RNAs were not observed (Fig 1C) [36]. Taken together, we concluded that the exosomal fraction was enriched in exosome-like vesicles.

### An overview of the RNA sequencing results

On an average, approximately 49 and 50 million reads were obtained from three independent biological replicates of the exosomal fraction and MGCs. Of those, about 33 million (67.52%) and 38 million (77.68%) paired reads were mapped to the porcine genome, respectively (S2 Table). Among the mapped fragments, approximately 7 million (43.17%) of the exosomal fraction and 12 million (62.70%) of MGCs were mapped to exons of known porcine transcripts. A total of 14,195 and 13,502 transcripts were detected in the exosomal fraction and MGCs, respectively, at an RPKM threshold of more than 0.1. Detailed information of independent samples is summarized in S2 Table.

### Abundant mRNAs in the exosome-like vesicles of pFF

The top 30 annotated mRNAs in the exosomal fraction and MGCs are shown in Tables 1 and 2, respectively. *EEF1A1* was the most abundant transcript in the exosomal fraction that also showed high abundance in MGCs. While the transcripts encoding ribosomal proteins (ribosomal proteins of the large subunit [RPL] and ribosomal protein of the small subunit [RPS]) were preferentially detected in the exosomal fraction, these transcripts were less abundant in MGCs. FAU, ubiquitin-like and ribosomal protein S30 fusion (*FAU*) and ubiquitin A-52 residue ribosomal protein fusion product 1 (*UBA52*) detected in the exosomal fraction also encode ribosomal protein-related products [37]. Therefore, the transcripts enriched in the follicular exosome-like vesicles seemed to encode ribosomal proteins, consistent with the results reported in human salivary exosomes [38].

**Table 1:** Top 30 most abundant mRNAs in exosome-like vesicles in pFF.

Gene symbol	Ensemble ID	RPKM (Exosomal Fraction)	RPKM (MGC)
*EEF1A1*	ENSSSCG00000004489	5,088.21	4,271.57
*RPS27*	ENSSSCG00000006558	4,345.22	2,565.47
*RPL34*	ENSSSCG00000003930	3,173.29	1,406.04
*RPS7*	ENSSSCG00000027353	2,684.54	1,086.50
*RPS3*	ENSSSCG00000014855	2,468.42	935.21
*RPL13A*	ENSSSCG00000003166	2,411.18	909.15
*RPS12*	ENSSSCG00000004177	2,384.40	1,187.95
*RPL31*	ENSSSCG00000008170	2,326.62	921.19
*RPS20*	ENSSSCG00000006249	2,321.79	1,211.61
*RPS23*	ENSSSCG00000014133	2,297.01	1,073.43
*RPS16*	ENSSSCG00000020817	2,250.29	721.35
*RPL18*	ENSSSCG00000025928	2,163.01	857.09
*RPS18*	ENSSSCG00000001502	2,123.72	1,140.27
*FTH1*	ENSSSCG00000014540	2,101.05	1,120.50
*UBA52*	ENSSSCG00000013907	2,070.60	1,068.80
*FAU*	ENSSSCG00000013002	2,051.53	791.49
*RPL35*	ENSSSCG00000005595	1,924.86	744.56
*RPL34*	ENSSSCG00000009146	1,918.96	726.29
*RPL36*	ENSSSCG00000030010	1,884.65	861.65
*RPS28*	ENSSSCG00000013597	1,873.97	853.43
*RPLP1*	ENSSSCG00000004970	1,824.04	1,038.76
*RPLP2*	ENSSSCG00000012842	1,821.14	809.35
*RPL23A*	ENSSSCG00000017768	1,811.04	1,241.32
*RPL13*	ENSSSCG00000024974	1,785.49	578.25
*RPS9*	ENSSSCG00000029785	1,703.30	565.85
*ACTG1*	ENSSSCG00000028355	1,698.27	1,325.06
*RPL17*	ENSSSCG00000029642	1,689.39	690.23
*FTL*	ENSSSCG00000003153	1,644.42	428.01
*RPS25*	ENSSSCG00000015103	1,615.55	1,034.76
*RPL5*	ENSSSCG00000006899	1,612.40	735.82

**Table 2:** Top 30 most abundant mRNAs in porcine MGCs.

Gene symbol	Ensemble ID	RPKM (Exosomal Fraction)	RPKM (MGC)
*COX3*	ENSSSCG00000018082	1,248.76	5,485.29
*COX2*	ENSSSCG00000018078	919.28	4,994.62
*EEF1A1*	ENSSSCG00000004489	5,088.21	4,271.57
*COX1*	ENSSSCG00000018075	980.54	3,863.89
*ATP6*	ENSSSCG00000018081	861.08	3,401.24
*RPS27*	ENSSSCG00000006558	4,345.22	2,565.47
*SERPINE2*	ENSSSCG00000016233	239.34	2,449.40
*INHA*	ENSSSCG00000020771	624.58	2,377.75
*GPX3*	ENSSSCG00000017092	1,097.51	2,221.39
*RPS8*	ENSSSCG00000003930	3,173.29	1,406.04
*ACTG1*	ENSSSCG00000028355	1,698.27	1,325.06
*CYTB*	ENSSSCG00000018094	277.78	1,284.82
*VIM*	ENSSSCG00000011033	1,254.67	1,281.73
*RPL23A*	ENSSSCG00000017768	1,811.04	1,241.32
*RPS20*	ENSSSCG00000006249	2,321.79	1,211.61
*RPS12*	ENSSSCG00000004177	2,384.40	1,187.95
*ACTB*	ENSSSCG00000007585	1,005.93	1,164.69
*RPS18*	ENSSSCG00000001502	2,123.72	1,140.27
*INHBA*	ENSSSCG00000021865	96.79	1,135.76
*FTH1*	ENSSSCG00000014540	2,101.05	1,120.50
*ENO1*	ENSSSCG00000022343	758.20	1,111.73
*RPS7*	ENSSSCG00000027353	2,684.54	1,086.50
*RPS23*	ENSSSCG00000014133	2,297.01	1,073.43
*UBA52*	ENSSSCG00000013907	2,070.60	1,068.80
*ND3*	ENSSSCG00000018084	240.18	1,039.76
*RPLP1*	ENSSSCG00000004970	1,824.04	1,038.76
*RPS25*	ENSSSCG00000015103	1,615.55	1,034.76
*CALR*	ENSSSCG00000013746	189.64	1,005.75
*RPS29*	ENSSSCG00000005003	944.30	978.18
*RARRES1*	ENSSSCG00000028623	116.73	970.41

The other highly abundant mRNAs in the exosomal fraction were ferritin heavy chain 1 (*FTH1*), actin gamma 1 (*ACTG1*), and *FTL*, all of which also showed high expression in MGCs. As for MGCs, cytochrome c oxidase 3 (*COX3*) was the most abundant mRNA, and *COX2* and *COX1* were highly expressed. These COX transcripts were also detected in the exosomal fraction at a relatively high levels (RPKM of 980.54, 919.28, and 1248.76 for *COX1*, *COX2*, and *COX3*, respectively).

### Detection of full-length mRNAs in the exosome-like vesicles

**Fig 2:**
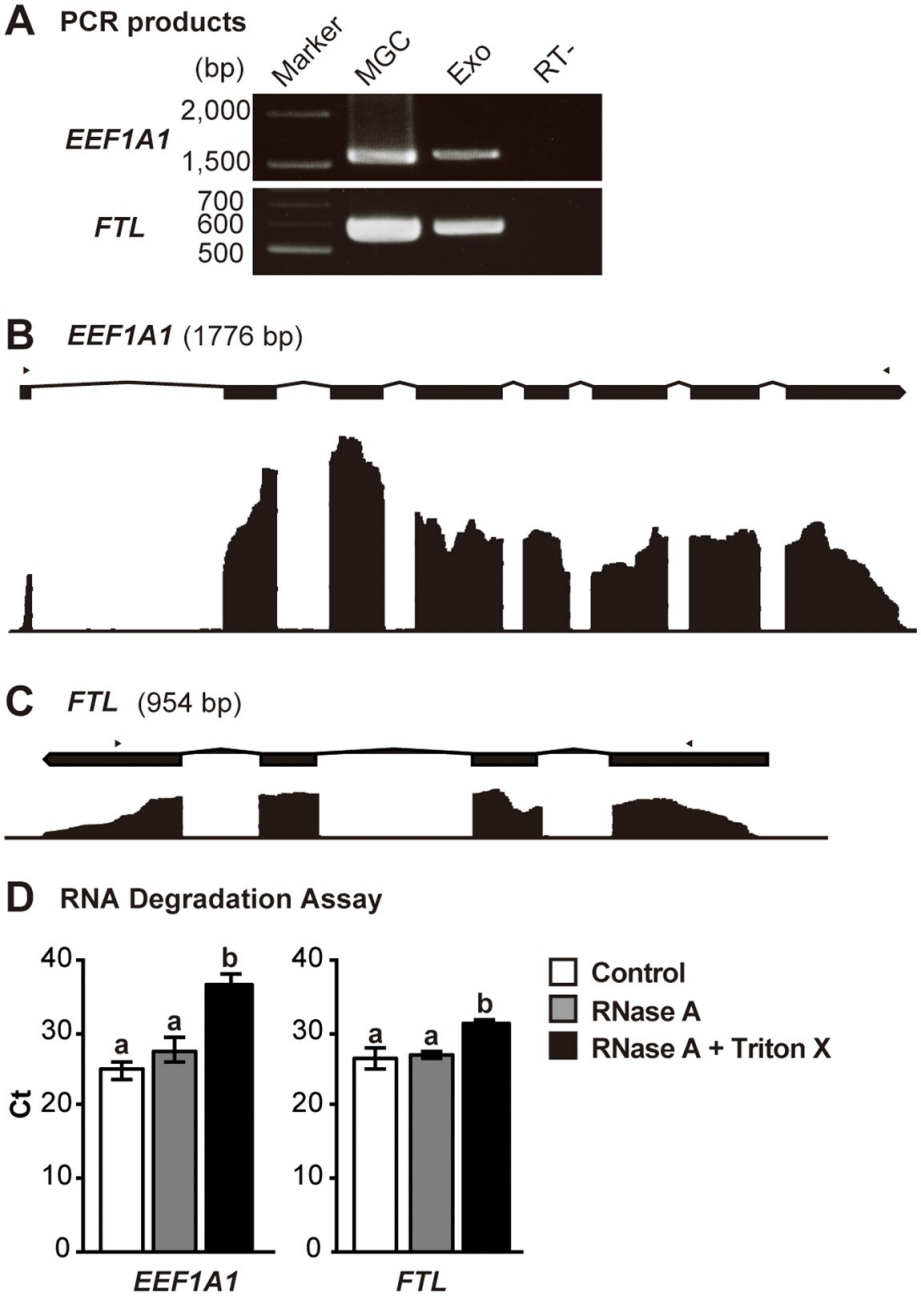
Genomic view of *EEF1A1* and *FTL* genes along with the representative RNA sequencing results of the exosomal fragments and RNA degradation assay. (A) RT-PCR analyses for *EEF1A1* and *FTL*. Marker, electrophoresis marker; Exo, exosomal fraction; MGC, mural granulosa cells. (B) *EEF1A1* and (C) *FTL* genes (upper panels) are shown in exons (black squares) and introns (polygonal lines), and the representative RNA sequencing results for each position in the genes are shown in a coverage graphs (lower panels). Arrowheads indicate positions of PCR primers used for RT-PCR shown in (A). RNA degradation assay using RNase A and Triton X-100. pFF was treated with RNase A (gray bars) with/without Triton X-100 (black bars) or PBS (control; white bars). The Ct values of total RNA extracted from the exosomal fractions were compared among these groups. Values with different letters (a and b) are significantly different (P <0.05) (n = 4).

We tested whether follicular exosome-like vesicles carry full-length mRNAs. As shown in Fig 2A, the bands with the expected sizes of *EEF1A1* and *FTL* were detected in the exosomal fraction by RT-PCR after the amplification of the regions between the first and the last exons of these transcripts. In addition, the RNA sequencing results showed high coverage of all exons of *EEF1A1* and *FTL* transcripts (Fig 2B and 2C). Moreover, to confirm that these RNAs are confined within the vesicles, RNase A and detergent (Triton X-100) treatment of the vesicles was performed. As shown Fig 2D, the Triton X-100 with RNase A treated group exhibited significantly higher Ct value than the RNase A treated and control groups. On the other hand, there were no significant differences between the control and RNase A-treated groups. These results indicate that RNAs are confined within the vesicles and protected from exogenous RNase activity. These results suggest that the follicular exosome-like vesicles contain full-length mRNA, and therefore, the mRNAs transferred by the vesicles may be translated into proteins and affect the biological processes of recipient cells as was reported previously [2, 21].

### Functional analysis of mRNAs in the exosome-like vesicles

**Fig 3:**
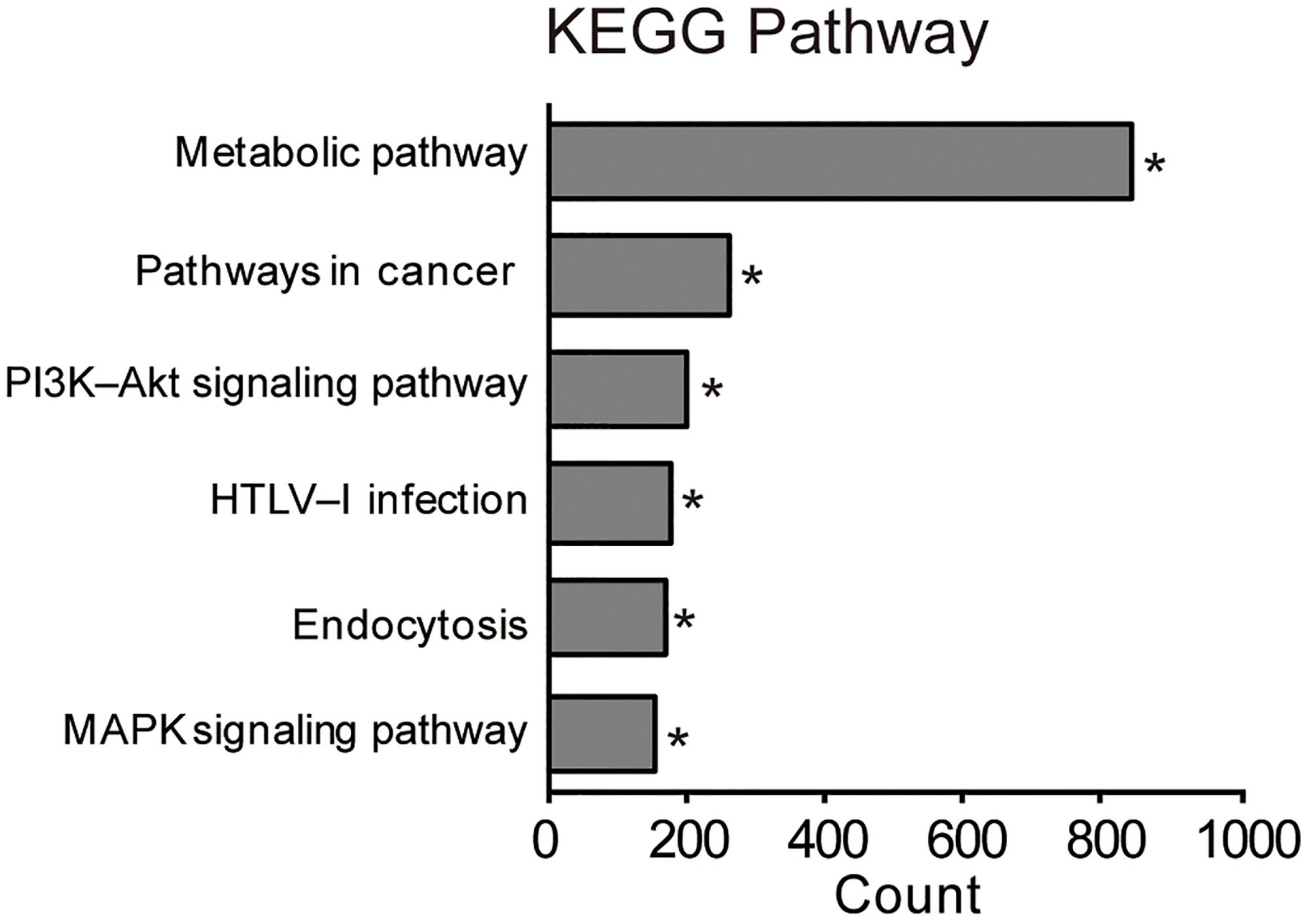
KEGG pathway analysis on the mRNA profile of exosome-like vesicles. The top six pathways are shown. For each pathway, bar plots show the counts of genes that belong to the pathway. Asterisk denotes the biological significance (P < 0.05).

Given that follicular exosome-like vesicles contain full-length mRNAs that may be translated to affect biological processes in recipient cells, we performed the Kyoto Encyclopedia of Genes and Genomes (KEGG) pathway enrichment analyses using DAVID (version 6.8) [25] and evaluated the potential effects of the vesicle-transferred mRNAs on recipient cells. In this analysis, 11,304 transcripts were functionally annotated with DAVID. As shown in Fig 3, the KEGG pathway analysis showed that the mRNA involved in “metabolic pathway”, “pathways in cancer”, “phosphatidylinositol-4,5-bisphosphate 3-kinase (PIK3K)-protein kinase B (AKT) signaling pathway”, “human T-lymphotropic virus (HTLV) infection”, “endocytosis”, and “MAPK signaling pathway” were enriched in the fraction. Therefore, the follicular exosome-like vesicles may affect these pathways in recipient cells.

### Comparison of transcriptomes between the exosome-like vesicles and MGCs

**Fig 4:**
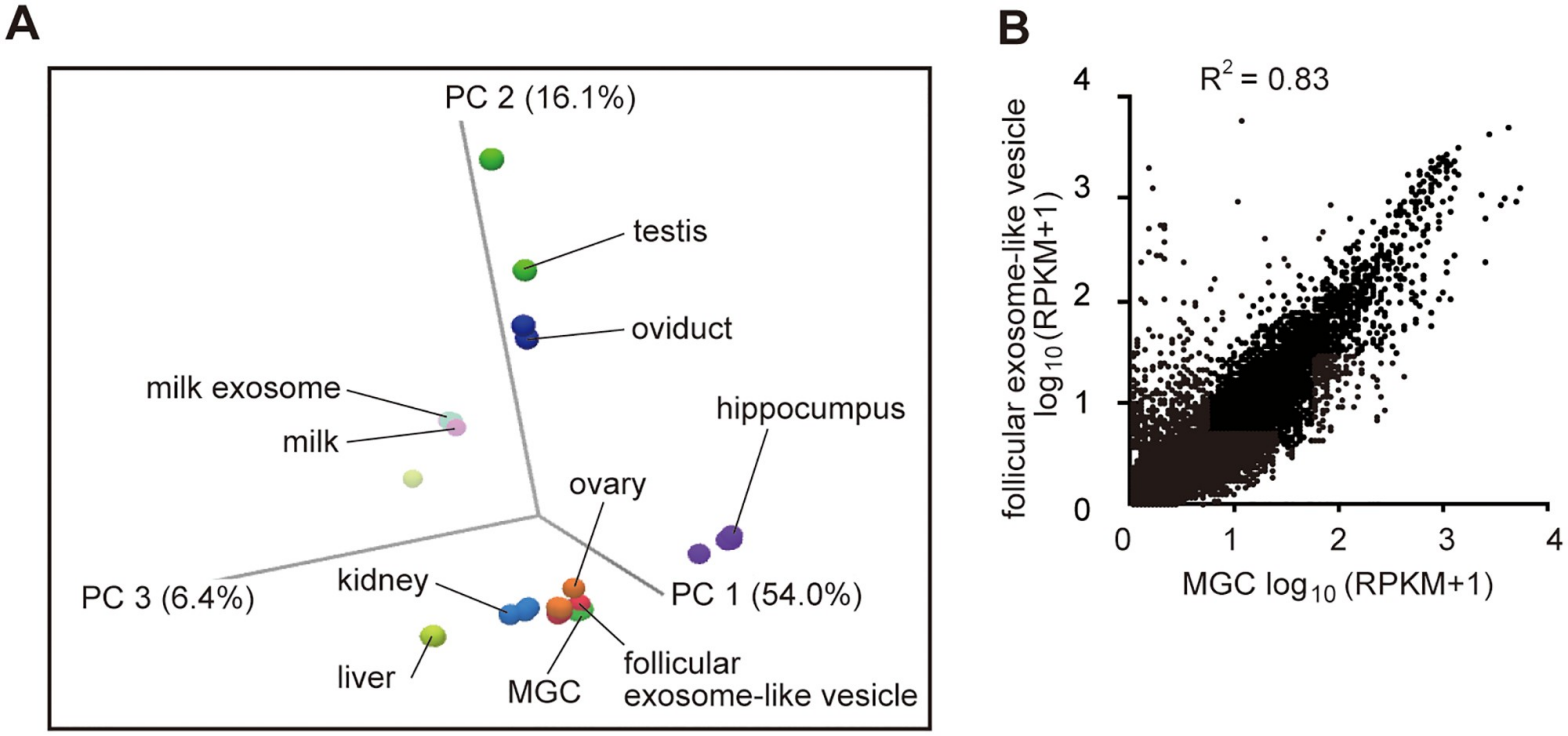
Comparisons of the mRNA profiles between exosome-like vesicles and mural granulosa cells (MGCs) in porcine follicles. (A) Principle component analysis (PCA) of the mRNA profiles of the follicular exosome-like vesicles and MGCs together with other porcine organs (see S1 Table for detail). (B) Scatter plot comparison of mRNA profiles of follicular exosome-like vesicles and MGCs.

To examine the similarity of the mRNA profiles between exosome-like vesicles and MGCs, PCA was conducted (Fig 4A). Aside from the mRNA profiles of exosome-like vesicles and MGCs, those of various porcine tissues available from NCBI GEO database were used (S1 Table). As shown in Fig 3A, exosome-like vesicle samples were clustered considerably close to those of MGCs and ovary samples than to other tissue samples. Moreover, the levels of transcripts were well correlated between exosome-like vesicles and MGCs (R^2^ = 0.83) (Fig 4B). Therefore, the mRNAs profile of exosome-like vesicles resembled that of MGCs, and MGCs are likely to be the main source of follicular exosome-like vesicles. However, it is important to note, several transcripts were exclusively detected in the exosomal fraction by RNA sequencing (see below for detail), suggesting that some of the follicular exosome-like vesicles may be secreted from cells other than MGCs.

### Unique mRNAs contained in exosome-like vesicles

Although the mRNAs profile of the exosomal fraction resembled that of MGCs, it carried mRNAs that were barely detectable in MGCs (Fig 4B). This observation suggests that the fraction may contain vesicles secreted from the cells other than MGCs. To identify the potential origin of follicular exosome-like vesicles other than MGCs, we focused on these transcripts. We used strict criteria to identify such transcripts; i.e., mRNAs from exosomal fractions with RPKM value > 10 and undetected in MGCs (RPKM < 0.1).

With this criteria, 14 mRNAs were identified as the unique transcripts in the exosomal fraction (Table 3). Some of the mRNAs are known to be specifically expressed in organs/tissues, including skeletal muscle, heart, liver, brain, and kidney [39–43]. These results suggest the possibility that a part of these exosome-like vesicles may be derived from non-ovarian organs/tissues through blood stream.

**Table 3:** Unique mRNAs in exosome-like vesicles undetected in MGCs.

Gene name	Ensemble ID	Exosomal Fraction (RPKM)	High expression tissue
*LOC100628118*	ENSSSCG00000025858	33.60	–
*FXYD1*	ENSSSCG00000021374	28.15	skeletal muscle, heart
*A2M*	ENSSSCG00000000660	26.54	liver
*VSNL1*	ENSSSCG00000008615	21.48	brain
*COL26A1*	ENSSSCG00000007678	14.92	–
*C1QTNF5*	ENSSSCG00000024936	14.77	retina
*LCN2*	ENSSSCG00000005638	14.63	trachea, bone marrow, lung
*CLDN5*	ENSSSCG00000010123	13.07	lung
*POU5F1*	ENSSSCG00000001393	13.06	colon
*ARX*	ENSSSCG00000020801	11.96	ovary
*LOC100737651*	ENSSSCG00000023783	11.06	–
*LMCD1*	ENSSSCG00000011538	11.00	skeletal muscle, lung
*ADGRA2*	ENSSSCG00000015825	10.72	small intestine, thymus, colon
*UMOD*	ENSSSCG00000007859	10.09	kidney

## Discussion

In this study, we performed the transcriptome analysis of the exosome-like vesicles in porcine ovarian follicles and focused on mRNA profiles. While the miRNA contents in exosome-like vesicles from ovarian follicles have been examined in detail [10, 16, 44–46], to the best of our knowledge, this is the first report to investigate the mRNA profiles of the exosome-like vesicles in ovarian follicles. The present analysis revealed that these vesicles contain various mRNAs which are enriched in those encoding ribosomal proteins. Moreover, bioinformatics analysis demonstrated that mRNA involved in the biological pathways known to important for normal follicular development were enriched in the exosome-like vesicles. While the majority of the vesicles are likely to be secreted from MGCs, some of these vesicles originated from non-ovarian organs/tissues. Therefore, the present study has unveiled several novel features of exosome-like vesicles in ovarian follicles by focusing on mRNA profiles.

The mRNAs detected in the follicular exosome-like vesicles are predicted to affect biological pathways such as metabolic, PI3K-AKT, and MAPK signaling pathways in the recipient cells. The precise control of the metabolic pathways such as lipid metabolism, glycolysis, and cholesterol biosynthesis is critical for the normal development of follicles and oocytes [47–49]. The PI3K-AKT signaling pathway is known be a critical regulator of quiescence, activation and survival of primordial follicles [50], proliferation and differentiation of granulosa and thecal cells [51], and meiotic maturation of oocytes [52]. Moreover, MAPK signaling pathway is known to be involved in steroid genesis, and oocyte maturation [13, 53, 54]. Although further studies which will test whether the transferred-mRNAs are translated into proteins in recipient cells are warranted, exosome-like vesicles may be involved in the control for the normal development of follicles and oocytes through the regulation of these pathways via mRNA transfer.

The promotive/supportive effects of exosome-like vesicles on the expansion of cumulus cells have been reported in cows and pigs [18, 19]. As normal cumulus expansion requires the activation of MAPK signaling pathway [54] and gastric cancer exosomes promote the tumor cell proliferation via MAPK signaling pathway activation [55], exosome-like vesicles may exert their effects on cumulus expansion through the transfer of mRNAs involved in the control of MAPK signaling pathway.

Some unique transcripts in exosome-like vesicles and barely detectable in MGCs were identified. A possible explanation is that the exosome-like vesicles may be secreted not only from MGCs but also from other ovarian cells such as oocytes, theca cells, ovarian interstitial cells, and non-ovarian organs/tissues. This hypothesis may be supported by the detection of *CYP17A1* mRNA, known to be highly expressed in theca cells, in the exosomal fraction (RPKM = 4.23) but not in MGC samples (RPKM < 0.1). Oocyte-specific transcripts such as *ZP2* and *ZP3* were detected in the exosomal fraction (RPKM 0.30 and 3.22, respectively), suggesting that the oocyte-derived exosomes may exist in the follicular fluid. This possibility is supported by the presence of oocyte-derived vesicles within the perivitelline space of mouse oocytes [56]. In addition, several mRNAs known to be expressed in non-ovarian organs/tissues were detected in the exosomal fraction, suggestive of the presence of the vesicles from non-ovarian organs/tissues. Another possible origin of exosome-like vesicles is that the vesicles secreted during early periods of follicular development or gonadal stage may remain until the formation of antral follicles. This hypothesis may be supported by the detection of *POU5F1* (known as OCT4) (RPKM = 13.06), known to be expressed highly at the gonadal stage, in the exosomal fraction. Further studies characterizing individual particles of follicular vesicles may clarify these possibilities.

Although the present study suggests that a part of the exosome-like vesicles present in follicular fluid may be supplied via blood from other organs/tissues, several questions remain to be answered. First, great care was taken to prevent contamination of the follicular fluid sample with blood, but the results of this study do not completely deny the possibility of contamination of our samples with blood. Second, the effects of exosome-like vesicles from other tissues on ovarian function are yet undetermined. Recent studies have showed that the adipose tissue-derived exosomes modulates insulin sensitivity in the liver and muscle tissues [57] and regulate gene expression in the liver [58]. Thus, a similar mechanism may exist between the ovary and other organ/tissues; i.e., non-ovarian organ-derived exosome may affect ovarian functions. However, further studies testing this possibility are warranted.

In summary, this study identified the characteristics of the mRNA transcriptome of exosome-like vesicles from follicular fluids of pigs. Bioinformatics analyses demonstrated that the mRNAs contained in the vesicles potentially modulate the signaling pathways involved in folliculogenesis. While most of exosome-like vesicles in the follicular fluid are likely to have originated from MGCs, our results suggest the presence of vesicles in the follicles from organs other than ovaries. Further functional investigations based on these results may help us to understand the physiological roles of exosome-transferred mRNAs in the regulation of ovarian functions.

## Supporting information

S1 Table List of the data sets used for the principle component analysis (PCA). (XLSX)

S2 Table Summary of the numbers of mapped reads. (XLSX)
